# Quasi-monolithic mirror suspensions in ground-based gravitational-wave detectors: an overview and look to the future

**DOI:** 10.1098/rsta.2017.0281

**Published:** 2018-04-16

**Affiliations:** SUPA School of Physics and Astronomy, University of Glasgow, Kelvin Building, Glasgow G12 8 QQ, UK

**Keywords:** mirror, suspensions, quasi-monolithic, silica, sapphire, silicon

## Abstract

At the commencement of a new era in astrophysics, with added information from direct detections of gravitational-wave (GW) signals, this paper is a testament to the quasi-monolithic suspensions of the test masses of the GW detectors that have enabled the opening of a new window on the universe. The quasi-monolithic suspensions are the final stages in the seismic isolation of the test masses in GW detectors, and are specifically designed to introduce as little thermal noise as possible. The history of the development of the fused-silica quasi-monolithic suspensions, which have been so essential for the first detections of GWs, is outlined and a glimpse into the status of research towards quasi-monolithic suspensions made of sapphire and silicon is given.

This article is part of a discussion meeting issue ‘The promises of gravitational-wave astronomy’.

## 1. Introduction

The first detection of a gravitational-wave (GW) signal by the Advanced LIGO (aLIGO) GW detectors in September 2015 [1] was a huge milestone for the field of physics. It not only confirmed through direct observation the prediction by Albert Einstein [2] that such waves should exist, but also gave the first direct observation of black holes through the ripples in space–time, produced as two black holes spiralled together to coalesce and form one larger black hole. Further binary black-hole mergers have been observed since [3,4], and the inclusion of Advanced Virgo in the network has greatly improved the directional resolution [5]. To top that, the exciting new era of multi-messenger astronomy has begun with the recent discovery of GWs from merging binary neutron stars [6] and coincident observations in the electromagnetic spectrum by the astronomy community [7], giving a first overarching picture of the processes involved during and after a neutron star merger.

This paper is aimed to be, as well as a glimpse to the future, a testament to the many years of research and development of a vital part of the ground-based interferometric GW detectors that are now observing GW signals: the quasi-monolithic fused-silica pendulum suspensions of the test masses.

In any type of GW detector, the aim is to freely suspend the test masses such that their motion corresponds to free-falling masses within the local gravitational field. In detectors based on Earth, one cannot escape from the influence of its gravitational field, so one has to suspend the test masses in some way, supporting against gravity but enabling free movement in all other degrees of freedom. Also the test masses should be isolated from external disturbances, in particular seismic noise (external vibrations transmitted through the ground and infrastructure). In GW detectors this is typically achieved in two broad stages: (i) a seismic isolation platform/system; and (ii) a pendulum suspension system of each test mass (as this acts as an effective mechanical filter in the horizontal direction above its resonance frequencies). The seismic isolation that can (and must) be achieved at frequency *f* = 10 Hz in this way is a factor of approximately 10^10^ with background seismic motion of order 10^−7^ to 10^−8^/*f* ^2^ m/√Hz in the sensitive direction [8–12]. Using this method an approximately 10^−22^/√Hz strain of space time produced on Earth by a GW (such as a binary black hole merger 1 billion light years away) can be detected.

Thermal noise is another important noise source in GW detectors between 10 and 100 Hz, which is the reason pendulum suspensions are chosen for the final stage. Each atom in the material used for the suspensions and test mass mirrors has some thermal energy and, thus, some associated thermally driven motion. The dominant sources of thermal noise are Brownian noise and thermo-elastic noise arising from the different parts of the final stage of the suspension and test mass [13]. This includes noise arising from the fibres, any joints, test mass and the coatings. These can be minimized over a broad frequency spectrum by designing the suspension such that it has as little mechanical loss, ‘damping’, which is directly related to thermal noise, as possible.

Pendulum suspensions exhibit intrinsically low mechanical loss. As most of the energy in a pendulum is stored in the dissipationless local gravitational field, there is ‘dissipation dilution’ in the pendulum direction [14,15], meaning that the mechanical loss of the pendulum can be orders of magnitude lower than the intrinsic material loss of the wires/fibres used to suspend a pendulum mass.

However, even using this, thermal noise can be a noise source that can limit detector sensitivity. Therefore, reducing thermal noise of pendulum suspensions has been a key topic of research [16,17]. These suspensions have evolved to become quasi-monolithic multi-pendulum suspensions to minimize thermal noise, and a wealth of research has been conducted and continues into developing the required technologies. In the following sections, we will focus on fused-silica suspensions installed at the time of the GW detections and their development history. We will then review the status of research for upgrades to the current detectors as well as future generations of detectors.

## 2. History of the development of fused-silica mirror suspensions

**Figure 1.**
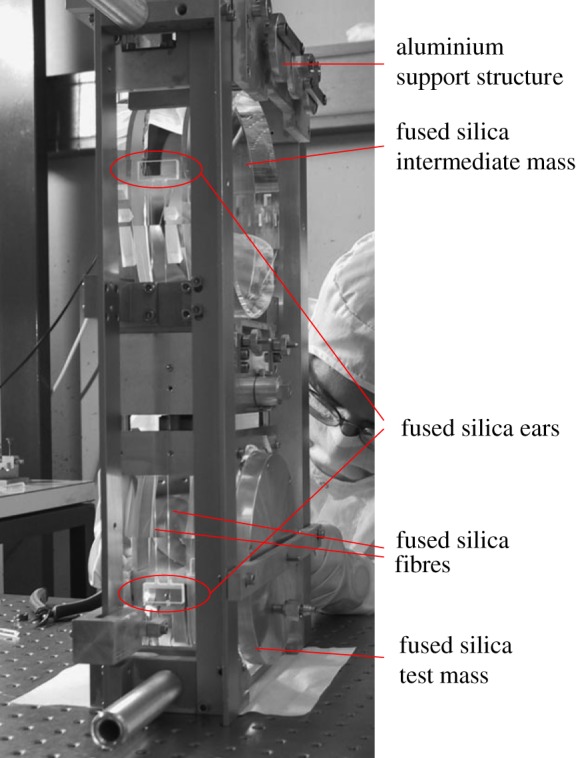
A GEO600 quasi-monolithic mirror suspension [39], showing the fused-silica ears bonded to the sides of the fused-silica penultimate and test masses with fused-silica fibres welded to horns on the ears.

Interferometric GW detectors came into play internationally in the late 1970s to the early 1980s with prototype GW antennae such as the Glasgow 10 m [18], the Garching 30 m [19] and Caltech 40 m interferometers [20]. The test masses in these interferometers were suspended as pendulums on metal wires (typically steel music wire) looped around the barrel. The thermal noise arising from the wires and the friction at the break-off points was deemed low enough to be implemented in the design and construction of the long baseline detectors LIGO and Virgo. These both used steel suspension wires (music wire) [21–23]. At this stage, the thermal noise contribution from the wires for the 4 km long arms of the LIGO detectors was still below the photon shot noise [13] limits, and a strain sensitivity of 1 × 10^−19^/√Hz at 100 Hz could be achieved. It was clear, however, that for any improvements in sensitivity to LIGO and Virgo to be achieved, alternative solutions to wire suspensions were needed. In fact for GEO600, with its shorter 600 m arm length, it was determined that thermal noise from metal wires would limit the sensitivity of the detector. Therefore, steps were taken to find alternative wire materials [24], before finally settling on fused silica, which had more than a factor 100 lower mechanical loss than any metal wires tested [25,26]. The thermal noise contribution from the suspension wires/fibres could in that way be pushed below 1 × 10^−19^/√Hz at 50 Hz [26–29]. A considerable research and development effort was required to make sure silica fibres could be produced that were strong enough and therefore thin enough to produce sufficiently low bounce mode frequencies [28]. The silica fibres for GEO600 were produced with a hydrogen–oxygen flame pulling machine enabling the bounce frequency to be repeatable to within 3.1% [30]. The bounce frequency requirement follows from mass position and angle tolerances. This in turn helps to put constraints on the fibre diameter and length. Also the fused-silica fibres needed to be attached to the test mass instead of being looped around in the same way as wires, as micro-cracks in silica produced when the fibres are touched significantly reduce their strength. The solution was found in attaching fused silica pieces known as ‘ears’ onto the sides of the test masses using a technique called hydroxide catalysis bonding (HCB) and then ‘welding’ the fibres to horns on the ears. HCB, which had been invented and patented by Gwo [31,32] and was first implemented for the Gravity Probe B mission [33,34], also proved to remove friction losses. Significant effort went into testing the technique for its suitability (strength and thermal noise contribution) [35,36], showing that pendulum Qs as high as approximately 2 × 10^7^ could be achieved in such systems [37] and adapting the technique for GEO600 [38,39]. The silica fibres were interfaced with a penultimate mass in the same way. These ‘quasi-monolithic’ suspensions were successfully implemented in the GEO600 detector, which has now been in operation for more than 15 years. The penultimate mass in turn was suspended with steel music wire on a third isolation stage with steel blade springs to provide additional vertical isolation, making this a triple suspension with a quasi-monolithic fused-silica final stage [28,37–40] as shown in figure 1. The quasi-monolithic suspensions of GEO600 were one reason why GEO600 had similar sensitivity to the initial LIGO and Virgo detectors with their steel wire suspensions, despite its significantly shorter arm length.

**Figure 2.**
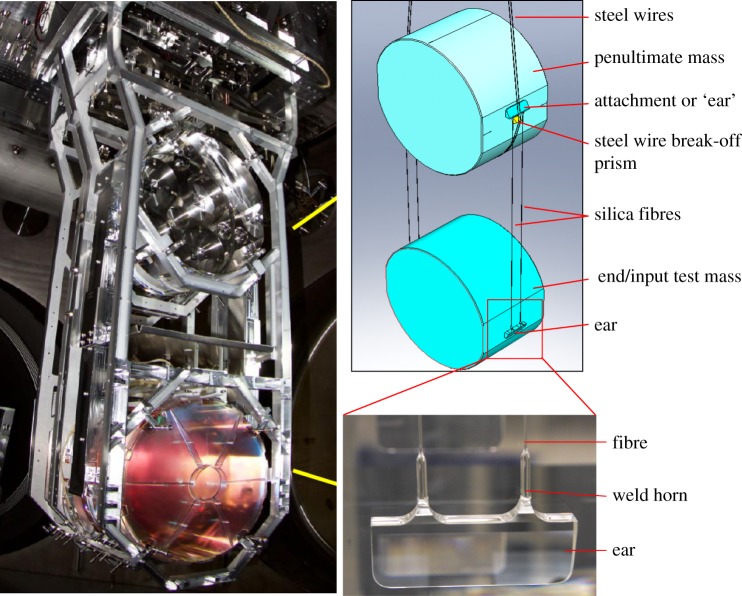
Photograph (left, LIGO-G1600324) and schematic of the aLIGO monolithic suspension (top right) with a photograph of an ear bonded onto the side of the test mass with fibres welded to it (bottom right).

**Figure 3.**
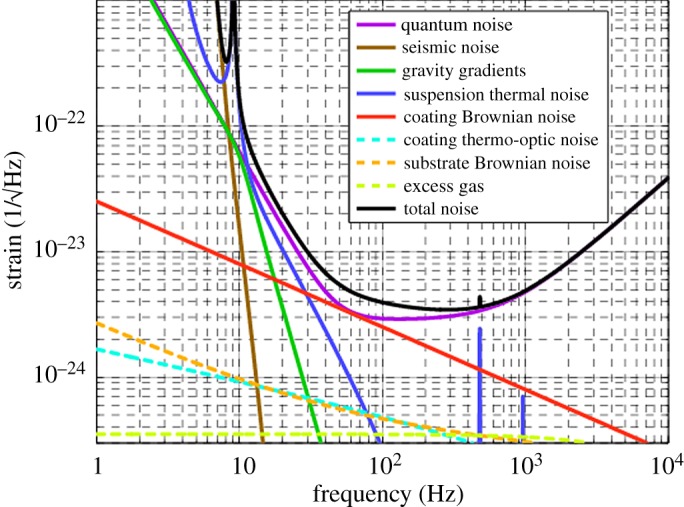
Noise budget of Advanced LIGO. This plot was produced using the GWINC (http://lhocds.ligo-wa.caltech.edu:8000/advligo/GWINC) and represents the Advanced LIGO broadband configuration. The blue curve represents the suspension thermal noise which is below other (limiting) noise sources from 10 Hz.

The idea of the fused-silica quasi-monolithic suspensions was quickly adopted as part of proposals for a major upgrade to LIGO, aLIGO [41], even before LIGO was fully operational. LIGO scientists knew that in order to improve sensitivity to 10^−19^ m/√Hz at 10 Hz, seismic isolation and thermal noise would have to be addressed. A quadruple suspension was proposed with a quasi-monolithic double pendulum (similar to GEO600) as the final two stages, and with three layers of cantilever springs above for vertical isolation. At that time sapphire test masses were considered the most promising approach (although in 2005 this was reconsidered and fused silica was selected as the test mass material [42]), and both circular and rectangular cross-section ‘ribbon’ fibres made of fused silica were considered. Further investigations into the parameters that influence the mechanical loss of silica fibres were conducted [43–46]. Development of silica fibre production also continued and big improvements were made by switching from fibres pulled from stock heated with a hydrogen–oxygen flame, to fibres pulled from stock heated with a CO_2_ laser beam. Fused silica absorbs light at 10 µm extremely well and, owing to its low thermal conductivity, can be heated locally very easily. The diameter and length of the laser pulled fibres could be controlled better as testified by the bounce frequency error of 0.8% [30] giving four times better control. The possibility of using ribbon fibres was explored for some time as the dilution factor could be made higher in this way (due to lower stiffness and thus higher compliance in the optical direction). However, making reliably strong ribbon fibres proved to be challenging, and it was found that the noise performance requirements could also be met by using dumbbell-shaped fibres. For these the thermal noise contribution from the welds could be reduced by allowing the fibre to bend far away from the weld, and the thermo-elastic noise could be reduced by choosing the diameter near the bending point such that the thermal expansion and Young's modulus terms cancel out [44,47,48]. The final design of the aLIGO quasi-monolithic suspension stage (shown in figure 2) is discussed in detail in [49]. This design is in many respects simply an upscaled version of the GEO600 suspension design. It has a 40 kg silica test mass and penultimate mass. Ears of very similar design to GEO600 are bonded on using the HCB technique with sodium silicate solution. The fibres are welded onto the ears with the masses *in situ* in the suspension structure, but, like the fibre production, the stock is now heated with laser light delivered to the welding site through a mirrored articulated arm, with which the welding area can be kept smaller and highly controlled. The development was supported by building five metal prototypes to evaluate mechanical behaviour, in-depth research into the strength of aLIGO fibres [50] and the assessment of expected thermal noise from the bonds [51]. It culminated in the installation of the final prototype, also including the quasi-monolithic fused-silica final stage, at the LASTI facility at MIT in 2010 [52]. With a few minor adaptations the quad suspensions with a monolithic final stage were then installed at the LIGO sites, and played a vital role in improving the seismic as well as thermal noise. The design sensitivity as well as noise budgets for aLIGO are shown in figure 3. With improvements to detector sensitivity still ongoing to reach design sensitivity, these suspensions perform extremely well, reaching violin mode Qs (the inverse of mechanical loss) of approximately 10^9^ [49], and, as with GEO600, dampers are applied to help with the control of the suspensions.

Virgo has also developed quasi-monolithic suspensions of a slightly different design [53], using the same CO_2_ laser technology to pull and weld fibres and using HCB for the jointing of interface pieces (albeit with a different procedure). Virgo in fact implemented this design before aLIGO and it became operational after intermediate upgrades, referred to as Virgo+ [54]. These monolithic suspensions contributed to improved sensitivity of the Virgo detector particularly in the low-frequency corner of its sensitive band in two observation runs between July 2010 and September 2011 [55]. This allowed for an improvement on the upper limit calculations of GW emissions from the Crab and Vela pulsars [56]. Following a delay in installing monolithic suspensions in the advanced configuration of the Virgo detector due to vacuum cleanliness issues [57], Virgo is currently installing its monolithic suspensions in time for the start of the next observation run in late 2018 [58,59].

## 3. Looking into the future: an overview of the current state of research

While the LIGO and Virgo scientific collaborations are currently working to get the advanced detectors to reach design sensitivity and to establish the new era of GW astrophysics and astronomy, there is also a focus on the future. The aim is to improve the sensitivity of the detectors even further, to allow for the possibility of detecting events further away and/or less powerful events, as well as observing continuous GW signals and studying the stochastic background. Both the possibility of building completely new detector facilities and improving the performance of current facilities are being considered, such as an upgrade of aLIGO to A+ in the short term [60]. LIGO Voyager is the term used for a mid-term upgrade to current detector facilities that would use cryogenic test masses [60] as thermal noise could be suppressed significantly this way. The Einstein Telescope (ET) [61] and Cosmic Explorer (CE) [62] are both considered for the long-term future, in different stages of development, which propose completely new facilities of larger scale and of which some elements could run cryogenically. ET is also proposed to be underground to reduce gravity gradient noise. The KAGRA detector [63] is currently being built and aims to have a sensitivity level comparable to that of the advanced detectors. It is championing both underground facilities and cryogenic test masses and suspensions.

This section focuses on the current status of the research towards these proposed detectors and detectors under construction.

### (a) Fused-silica mirror suspensions

Current proposals that involve room temperature operation and thus quasi-monolithic fused-silica suspensions include A+ [60] and one of the multiple detector systems in the ET [61]. For A+ this may involve small changes to the suspension to improve thermal noise performance such as thinner fibres and different ear design. For ET two types of detectors are proposed integrated on one site. It would have a low-frequency detector (ET LF), consisting of three cryogenic interferometers, and a high-frequency detector (ET HF), consisting of three room temperature interferometers, in a triangular configuration with arm lengths of 10 km in an underground facility [61]. The HF interferometers are proposed to use much heavier fused-silica test masses (up to 200 kg) than aLIGO with significantly longer fused-silica fibres. Research and development towards upgrades and new GW facilities, such as A+ and ET HF, is ongoing.

### (b) Cryogenic mirror suspensions

For cryogenic mirror suspensions, in which thermal noise power can potentially be suppressed as it is directly proportional to temperature, a change of test mass material is required because fused silica shows a broad increase in mechanical loss at lower temperatures and also has a low thermal conductivity. Instead, the main materials of interest are silicon and sapphire, as these materials show superior mechanical and thermal behaviour at cryogenic temperature. In general, the lower the temperature of operation the better is the thermal noise. For silicon this is true as the thermal expansion coefficient goes down to zero below 20 K. However, in silicon the thermal expansion coefficient also changes sign at 123 K and hence the thermo-elastic noise vanishes at this temperature. Therefore, for silicon test masses operation temperatures of both 123 K and below 20 K are of interest [61]. For sapphire an operating temperature below 20 K is preferred as the thermal expansion coefficient goes to zero at these temperatures. Research has been conducted on the possibility of building quasi-monolithic suspensions in silicon and sapphire since the Mid-1990s. Sapphire has attracted interest for a longer time as it was considered also as an optics material for room temperature operation in the LIGO detectors [41], and scientists in Japan have gained experience in producing sapphire suspensions for cryogenic GW detectors starting with experiments around 1998 [64]. However, silicon is proposed as the test mass material of choice for ET LF as its optical properties in the near-infrared region are well known and considered to be superior to those of sapphire. The history and current status of sapphire suspension development will be discussed first, followed by that of silicon mirror suspensions, which is comparatively in its infancy.

#### (i) Sapphire suspension development

Early papers, geared towards developing sapphire suspensions, focused on heat extraction through small-diameter sapphire fibres looped around a sapphire test mass [64], based on a requirement to dissipate tens of mW of absorbed laser power. They also addressed the mechanical loss of such fibres [65] and test masses [66]. However, as it has proved to be difficult to reduce the optical absorption of sapphire to below 100 ppm [67]; a higher heat extraction requirement of roughly 1 W has led to the development of much thicker (greater than 1 mm in diameter) sapphire fibres [68].

Around the same time planning started for building the Cryogenic Laser Interferometer Observatory (CLIO); a 100 m cryogenic Fabry–Perot interferometer was used in the Kamioka mine [69] to demonstrate the benefits of an underground and cryogenic facility to detector performance. CLIO uses sapphire test masses suspended on steel wires and demonstrated that a displacement sensitivity of 2.2 × 10^−19^ m/√Hz could be achieved at 17 K in 2012 [70,71].

**Figure 4.**
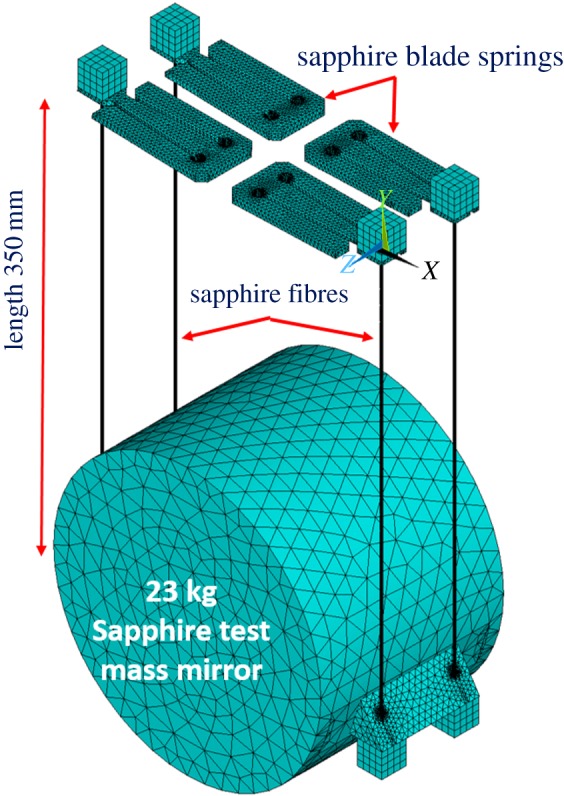
FEA (ANSYS) model of the KAGRA sapphire suspension system [82] (courtesy of Rahul Kumar, KAGRA).

Following this, the focus was directed to KAGRA, and to the development of ‘quasi-monolithic’ suspensions made of sapphire using the thicker sapphire fibres. One cannot loop these thicker fibres around a test mass, so methods of jointing sapphire to sapphire needed to be explored, potentially to work towards similar designs as used in GEO600, aLIGO and Advanced Virgo. HCB was an obvious option to explore, though it was not initially clear that making quasi-monolithic suspensions by bonding sapphire using HCB would be possible. This is due to the inert nature of sapphire, which will not react with hydroxide solutions if pristine [72]. Non-crystalline aluminium oxide does however react with KOH. Despite initially discouraging results [73], subsequent studies showed that bonding mechanically polished sapphire using HCB gives excellent strength results [74–76] both at room temperature and cryogenic temperatures, suggesting there is enough damage to the surface of the sapphire to allow the bond reaction to take place. The mechanical loss of a hydroxide catalysis bond (made with sodium silicate solution) was measured down to 7 K [77], showing the loss decreases with temperature to (3 ± 1) × 10^−4^ at 20 K. Also, it was demonstrated that the thermal conductance across a pair of sapphire rods joined using HCB was well above requirement for KAGRA [76]. In combination with findings on strength, this demonstrated that HCB was a suitable technique for the quasi-monolithic suspensions in KAGRA. One of the main concerns with jointing using HCB is that it is very difficult to recover the joint in any possible repair or replacement scenario. Therefore, as an alternative or complementary jointing technique for joints under compression, indium bonding was also investigated for KAGRA suspensions, as when used between a fibre and the ear, an indium joint can be removed relatively easily in case a repair is required. Therefore, in particular the mechanical loss [78,79] and heat flow across indium bonds were assessed [76,79,80]. For the latter work an indium joint was made between two sapphire cylinders by depositing an indium film on the bonding surfaces and then placing an indium foil between them and heating to 156°C under a mechanical load for 2 h. The loss measured was as low as 2 × 10^−3^ at 20 K and the thermal conductance through the layer was deemed not to limit the thermal heat extraction. The KAGRA mirror suspension is described in detail in [76,81]. It features machined sapphire fibres (1.6 mm diameter, 350 mm long) that are brazed (with a proprietary commercial technique) into highly polished blocks on each end (also called nail heads) [80]. As in GEO600, aLIGO and Advanced Virgo, an interface piece is bonded using HCB to two flat faces (which are not parallel) on the barrel of the test mass. The interface pieces have a triangular prism shape with slots through which the fibres run. The bottom nail heads are slotted underneath the interface pieces and jointed using indium bonding. At the top, the nail heads are slotted onto sapphire blade springs (that provide some more vertical isolation) which are in turn interfaced with the metal intermediate mass (figure 4). A test assembly of the sapphire suspension in one of the KAGRA cryostats developed for its cryogenic test mass suspensions [83] has now been made and a cooling test has shown the suspension can be cooled to 12 K in 23 days [63]. Gallium bonding instead of indium bonding was used for the nail head interfaces in this suspension [82]. Data on thermal conductance and mechanical loss have not been published as yet.

#### (ii) Silicon suspension development

Silicon needs to have an oxide layer to bond using HCB [84], so research has focused on studying the influence of the thickness of oxide layer and type of oxide layer on the strength [85,86]. The influences of a curing time of up to three weeks, surface flatness and crystal orientation of silicon have been studied [72], as well as the effect of thermal cycling on strength [87]. Work to understand the strength behaviour of hydroxide catalysis bonds in silicon is continuing. Some work has also focused on thermal conductance through [88] and the mechanical loss of bonds between silicon samples [89]. Further studies of this continue. Cumming *et al*. [90] reported on conceptual ribbon or round fibre designs looking at fibre dimensions set by requirements on the heat extraction capability of such fibres. They then calculated the off-resonance thermal noise associated with the loss of the fibres in a 200 kg silicon test mass suspended on four such fibres in a heat-conductance limited case for which as much as a factor of approximately 100 improvement could be achieved at 40 K with respect to aLIGO specification of approximately 1 × 10^−19^ m/√Hz at 10 Hz. They also conducted strength tests on silicon ribbons etched from silicon wafers as potential ribbon fibres. These were exposed to different surface treatments in an attempt to increase their strength, and averages of between 133 and 210 MPa for breaking stress were measured. Taking a safety factor of 3 on the lowest strength number for suspending a 200 kg test mass and ribbon fibres of 1 m in length, an improvement factor of 18 in thermal noise could be made, which is encouraging for designing silicon suspensions in the future. As this is the only research available in the literature at this point on silicon ribbon/fibre production, much more research is required. Furthermore, research on the development of the indium bonding technique for application to silicon suspension assembly and thermal conductance of silicon suspension elements and joints (both HCB and indium) is ongoing.

## 4. Conclusion

The development of quasi-monolithic suspensions was crucial to enabling the first detections of GWs and start of the era of GW astronomy. It took roughly nine years to the first implementation in GEO600 and another nine for installation in aLIGO. The technology involved includes production of fused-silica fibres at least as strong as steel of the same diameter using CO_2_ laser light, CO_2_ laser welding and HCB of ears. Combined, they have produced the lowest thermal noise test mass suspensions to date. Looking into the future, cooling the mirror suspensions to cryogenic temperatures using sapphire or silicon test masses is an attractive way forward for reducing the thermal noise yet further. KAGRA's sapphire suspensions have been under development since the late 1990s and also are of quasi-monolithic design involving HCB and indium (or gallium) bonding. The proposed silicon suspensions for LIGO Voyager, ET-LF and CE still have some way to go, particularly on fibre development. However, the development of jointing technologies (HCB and indium bonding) for silicon and initial tests with silicon ribbon fibres are proving to be fruitful lines of research.

## Data accessibility

This article has no additional data.
